# Reconciling the opposing effects of neurobiological evidence on criminal sentencing judgments

**DOI:** 10.1371/journal.pone.0210584

**Published:** 2019-01-18

**Authors:** Corey H. Allen, Karina Vold, Gidon Felsen, Jennifer S. Blumenthal-Barby, Eyal Aharoni

**Affiliations:** 1 Neuroscience Institute, Georgia State University, Atlanta, Georgia, United States of America; 2 Faculty of Philosophy, Leverhulme Centre for the Future of Intelligence, University of Cambridge, Cambridge, United Kingdom; 3 Department of Physiology and Biophysics, University of Colorado School of Medicine, Aurora, Colorado, United States of America; 4 Center for Medical Ethics and Health Policy, Baylor College of Medicine, Houston, Texas, United States of America; 5 Department of Psychology, Georgia State University, Atlanta, Georgia, United States of America; 6 Department of Philosophy, Georgia State University, Atlanta, Georgia, United States of America; University of Medicine & Dentistry of NJ - New Jersey Medical School, UNITED STATES

## Abstract

Legal theorists have characterized physical evidence of brain dysfunction as a double-edged sword, wherein the very quality that reduces the defendant’s responsibility for his transgression could simultaneously increase motivations to punish him by virtue of his apparently increased dangerousness. However, empirical evidence of this pattern has been elusive, perhaps owing to a heavy reliance on singular measures that fail to distinguish between plural, often competing internal motivations for punishment. The present study employed a test of the theorized double-edge pattern using a novel approach designed to separate such motivations. We asked a large sample of participants (N = 330) to render criminal sentencing judgments under varying conditions of the defendant’s mental health status (Healthy, Neurobiological Disorder, Psychological Disorder) and the disorder’s treatability (Treatable, Untreatable). As predicted, neurobiological evidence simultaneously elicited shorter prison sentences (i.e., mitigating) and longer terms of involuntary hospitalization (i.e., aggravating) than equivalent psychological evidence. However, these effects were not well explained by motivations to restore treatable defendants to health or to protect society from dangerous persons but instead by deontological motivations pertaining to the defendant’s level of deservingness and possible obligation to provide medical care. This is the first study of its kind to quantitatively demonstrate the paradoxical effect of neuroscientific trial evidence and raises implications for how such evidence is presented and evaluated.

## Background

Neuroscience is playing an increasing role in criminal trials. While it is unfeasible to estimate the prevalence of neurobiological evidence in lower courts, their rates in murder trials may exceed five percent, as indicated by the subset of cases documented at the appellate level [1]. But brain evidence can be complicated, raising questions about how fact finders interpret the quality of this evidence.

According to recent research, ordinary people have considerable preconceptions about the explanatory power of neurobiological evidence. Weisberg, Taylor, and Hopkins [2], for example, found that when lay people evaluate the quality of scientific explanations for behavior, their ability to distinguish between good and bad quality explanations was hampered by the presence of irrelevant neuroscience information. People judged explanations paired with the irrelevant neuroscience information as stronger and more satisfying than explanations without it. The investigators describe this context effect as evidence of the “seductive allure” of neuroscientific explanations. Furthermore, brain images, per se, may have a particularly persuasive impact on credibility judgments (e.g., [3]; but see [4–6]).

If people perceive evidence to be stronger when it is dressed up in neuroscientific garb, to what extent does this tendency impact legal judgments? In a study of trial court judges, Aspinwall, Brown, and Tabery [7], found that psychiatric testimony about a defendant’s mental illness reduced recommended prison sentence lengths when that testimony included a description of the illness’ biological causes. Similarly, in a mock trial study, Greene and Cahill [8] showed that in the case of high risk offenders, neuroscientific evidence of psychosis reduced the number of death sentence recommendations compared to the diagnosis alone. Likewise, Capestany and Harris [9] found that biological personality assessments reduced punishments compared to behavioral assessment in a mock trial study. Marshall and colleagues [10] found that neurobiological explanations reduced perceptions of dangerousness when the defendant is described at psychopathic. Finally, Shariff and colleagues [11] found that exposing people to general information about the neural bases of human behavior reduced the length of recommended prison sentences in mock trial cases.

One explanation for the apparent “seductive allure” of neurobiological evidence is that people assume that physical causes of behavior, such as genetic or neurological causes, indicate that the behavior is outside the agent’s ability to make free choices or to control their behavior and therefore outside their scope of responsibility. Thus, when physical causes are more salient than other causes, judgments should be more lenient. Consistent with this theory, Greene and Cohen [12] have argued that advances in neuroscience sow doubt about the causal roles of individual choice and control, and thus, the degree to which people should be held responsible for illicit actions.

Other scholars have argued that the tendency to excuse a person of responsibility simply because that behavior has identifiable physical causes is fallacious because, in reality, all actions are ultimately physically determined. Morse [13] names this fallacy the “fundamental psycholegal error,” and suggests to the contrary that there are legitimate reasons to hold people responsible for their actions even though those actions have physical causes (see also [14, 15]).

Fallacious or not, the inference that physical causes of a defendant’s behavior render that behavior outside his control carries another risk. Though such inferences can reduce attributions of responsibility, scholars have warned that they can potentially increase perceptions that the defendant is dangerous and in need of greater institutional supervision. For instance, Berryessa [16] found that potential jurors given evidence of biological risk factors rated the defendant as less responsible for their acts and more likely to commit future crimes compared to those not given biological risk factor information. This potential of biological evidence to cut both ways in a defendant’s case has been described as a double-edged sword [7, 17–19]. If admission of such evidence carries this risk, it has important implications for how legal parties present evidence, how judges and jurors evaluate that evidence, and more broadly how human beings make moral judgments.

Despite the growing number of studies on this topic, evidence of the double-edged nature of biological evidence has been elusive. Though biological evidence has tended to mitigate guilt and punishment in the aforementioned studies [7, 8], other studies have shown aggravating effects. For instance, McCabe, Castel, and Rhodes [20] found that potential jurors given incriminating fMRI lie detection evidence rendered more guilty verdicts than those given the same evidence in the form of a polygraph or thermal facial imaging test, as well as those given no lie detection evidence at all. In contrast to Greene and Cahill’s [8] observed effect of a reduction in death sentences, Saks, Schweitzer, Aharoni, and Kiehl [21] found that when neuroimaging evidence is proffered by the prosecution, death sentence recommendations increase.

Yet other mock trial studies have reported null effects of biological explanations. For example, neurobiological evidence of psychopathic or anti-social tendencies in criminal offenders had no effect on participant’s recommended prison sentence lengths compared to psychological or behavioral evidence [22, 23]. Similarly, Blakey and Kremsmayer [24] found that describing a case of aggravated assault as stemming from the offender’s impaired brain activity as opposed to his lower self-control had no significant impact on the length of prison sentence recommended for the crime. Finally, Schweitzer, Saks, Murphy, Roskies, Sinnott-Armstrong, and Gaudet [25] found that after conducting four experiments investigating the effects of neurobiological evidence on criminal sentencing, a meta-analysis demonstrated no effect of neurobiological evidence on guilt verdicts.

Despite expectations that neurobiological evidence cuts both ways, no single study has quantitatively demonstrated both mitigating and aggravating effects side by side. One reason could be that different studies have ignored potential attitudes about the disorder’s amenability to treatment. Highly treatable disorders tend to reduce perceptions that the defendant continues to be a danger to others. So if a biological disorder is portrayed as treatable, punishments should be lenient because the defendant will be perceived as low in both responsibility and dangerousness. But if the same disorder is portrayed as untreatable, this may evoke concerns that the defendant is dangerous even if he is less morally responsible for the crime. To our knowledge, no previous studies have considered the potentially moderating role of treatability.

Another possible reason for the inconsistent findings is that the dependent measures used across studies fail to capture distinct, sometimes competing, punitive motives within the same individual. Most quantitative studies, for instance, included only a single punishment measure, along the lines of: “How long should the offender be sentenced to prison?” or “How much should the offender be punished?”. If a participant has both deontological concerns (e.g., that the offender deserves to be punished for his moral transgression) and consequentialist concerns (e.g., that the offender must be incapacitated because he is a danger to society), she is forced to use a single measure to voice both types of concerns. When participants are forced to use prison time as a “one-stop shop” to capture their diverse punitive motives, these separate motives could interact or cancel out in unknown ways.

The present study was designed to address these limitations in a contrastive vignette experiment involving the diagnosis of an impulse control disorder following a sexual assault. A sexual assault crime was selected because it is one of the more plausible charges used to justify commitment to involuntary hospitalization in many U.S. states. By explicitly manipulating whether the neurobiological or psychological disorder is deemed treatable or untreatable, we control for the possible dependency of neurobiological descriptions on perceived treatability (see Ahn, Proctor, & Flanagan [26]). And, by providing participants with the option to sentence the offender to prison time (designed largely for moralistic punishment) and/or inpatient hospitalization time (designed largely for incapacitation but not punishment), we ensure that if the distinct punitive motives are evoked, they may be distinguished by a simultaneous reduction in prison and increase in involuntary hospitalization.

A supplemental goal of this project was to explore whether the predicted effects are associated with individual differences in cognitive functioning. Individual differences in legal settings are important because they can serve as sources of differential treatment of offenders. Leading theories suggest that many context effects are caused by a tendency to attend to the salient attributes and activate confirmatory associations in memory [27]. If so, it follows that people who excel in counterfactual reasoning (i.e., the tendency to entertain multiple possible perspectives or outcomes) should exhibit less susceptibility to context effects. Yet, other research suggests that individuals with high cognitive ability are no less susceptible to cognitive biases, and may even be even more susceptible in some cases [28]. Other theorists have emphasized the role of emotional regulation in context effects, suggesting that individuals who are better able to regulate emotions (for example, to reduce their emotional reactions to affective stimuli, such as pictures) are less susceptible to context effects due to a shift from an emotional strategy to a cognitive strategy [29]. If so, people with high emotion regulation ability should be less susceptible to the salience of neurobiological causes of behavior.

This study investigated the effect of brain-based evidence of an impulse control disorder on lay sentencing judgments in a large Internet-based sample. The use of lay samples to study judicial decision making is indirect at best. But lay samples are valuable for at least two other reasons: They are scientifically important to the extent that they help illuminate general patterns of human cognition, and they are legally important in that legal policy often relies on public opinion, as expressed through vehicles like the election of judges and legislators and the endorsement of ballot propositions and referenda. So, understanding punitive judgment formation among laypeople can help inform criminal punishment policies and practices even if these decisions don't necessarily generalize to judicial decision making.

The overarching rationale of this study was that if a neurobiological explanation of an impulse control disorder, more so than a psychological one, primes people to believe the defendant lacked control of his actions and should therefore be held less responsible for his crime, then such an explanation should result in reduced prison sentences. However, in this view, the same evidence should increase support for non-punitive custody (e.g., involuntary hospitalization) because a defendant who lacks control will also be perceived as more dangerous. This latter effect should be especially apparent when the defendant’s disorder is rendered untreatable. We thus tested the following hypotheses:

**H1.** Neurobiological evidence of a disorder will decrease prison sentences relative to psychological evidence and to no evidence.

**H2.** The H1 effect will be greater when the disorder is seen as treatable versus untreatable.

**H3.** Neurobiological evidence will increase involuntary hospitalization terms relative to psychological evidence and to no evidence.

**H4.** The H3 effect will be greater when the disorder is seen as untreatable.

**H5. (a)** The H1 effect will be best accounted for by a reduction in deontological concerns, and **(b)** the H3 effect will be accounted for by increased consequentialist concerns.

**EH1.** Individuals who score lower in executive functioning (counterfactual reasoning or emotion regulation) will exhibit decreases in punishment and involuntary hospitalization relative to higher scoring individuals.

## Methods

### Participants

Three hundred sixty nine adults residing in the U.S. (53% F, 47% M) were recruited from Amazon Mechanical Turk (MTurk) in November 2017 and paid $3.00 for participating. Thirty nine respondents were omitted for incomplete data, missed attention checks (e.g., “What colors are on the American flag?”), or for spending too little time on the survey (< 1 SD of mean, or ~5 minutes), resulting in a final sample size of 330. The average age was 36.0 years (*SD* = 11.38, range = 19–71). Median annual household income was $25,000-$49,999. Political party affiliation was 43.9% democratic, 29.0% independent, 15.5% republican, and 15.6% other. Human subject research was authorized by the Georgia State University institutional review board: H16349. Written consent was obtained from all participants.

The use of MTurk for research purposes has been documented elsewhere [30]. Like most sampling methods, use of the MTurk sampling pool presents some limitations on generalizability to the U.S. population, most notably in terms of political party affiliation, for which our sample disproportionately identified as democratic. However, this pool has been validated for research on political ideology [31]. More broadly, the MTurk sampling pool, and our sample in particular, are more representative on basic U.S. demographics, as defined by the 2017 U.S. Census, than other methods commonly employed in social science research such as the use of university students.

### Sample size estimation

The estimated sample size was determined by the power required to detect a significant interaction of mental health status (neurobiological vs. psychological) and treatability status (treatable vs. untreatable) on recommended punishment, assuming that the probability of obtaining a false positive is α = 0.05. Under this assumption, a sample of 327 participants provides 95% power to detect a significant interaction in this design where the effect size is f = 0.20 (a small effect size by conventional criteria; [32]).

### Design

The study used a 3 (Mental Health Status) x 2 (Treatability Status) incomplete factorial design with random assignment to conditions. Mental health status varied whether the defendant was described as having an impulse control disorder of neurobiological origins, of psychological origins, or was healthy. Treatability status varied whether the impulse control disorder was seen to be completely treatable or untreatable, but only for the neurobiological and psychological conditions, not the healthy condition. The primary dependent measures consisted of a preliminary, baseline prison sentence recommendation made before exposure to the manipulations, a revised prison sentence recommendation after the presentation of manipulations, and the amount of time the defendant should involuntarily spend in an inpatient hospital *after* the completion of his prison term (all from 0 to 4 yrs. as determined from unpublished pilot data). Change in prison sentence recommendation was calculated as the within-subject change from baseline—the difference between their baseline and revised prison sentence recommendation. In order to account for individual-level variation in punishment judgments, a composite punishment score was constructed, defined as the individual's revised sentence divided by his/her baseline punishment recommendation, yielding a percentage change score for each participant. Exploratory measures were designed to check and clarify the results of our hypothesis tests. These consisted of Likert-type ratings (from (1) “Strongly Disagree” to (7) “Strongly Agree”) for various statements regarding the defendant’s moral responsibility, blameworthiness, desert of punishment, free will, ability to stop himself, trustworthiness, danger to society, likelihood of reoffense, the degree to which the crime was an expression of his character, the perceived efficacy of treatment, and the perceived impact and importance of the evidence on their punishment decision.

### Materials

The case summary described an instance of sexual assault in which an adult male was found guilty of assaulting an adult female neighbor. (See S1 Appendix for stimuli.) Following receipt of this information, the participants were presented with a professional opinion regarding Mr. Edward’s mental status from either neurologists, psychologists, or “experts”—corresponding to our neurobiological, psychological, and healthy conditions, respectively. Participants were either informed that the neurologists had located a large tumor in the impulse control region of the defendant’s brain, that the psychologists had diagnosed the defendant with an impulse control disorder, or that the experts had determined that the defendant had no mental health issues. Within the neurobiological condition, participants were told that the neurologists either conducted surgery to successfully remove the tumor (treatable condition), or found it to be inoperable (untreatable condition). Similarly, those within the psychological condition were told that cognitive-behavioral therapy was either a success (treatable condition) or a failure (untreatable condition). Those within the healthy condition were given no further information. Aside from these manipulations across conditions, all participants received identical information.

Three additional measures were included after the dependent measures to assess individual differences. The Counterfactual Thinking (CFT) scale is used to assess the ability to reason counterfactually, as well as a measure of perspective switching and open mindedness—For example: “My beliefs would not have been very different if I had been raised by a different set of parents” [33]. The Difficulties in Emotion Regulation Scale-Short Form (DERS-SF) is meant to assess an individual’s ability to regulate his or her emotions [34]. Following these additional scales, participants self-reported his or her political ideology from (0) “very liberal” to (10) “very conservative”.

### Procedure

Participants were asked to complete the survey privately on their personal devices. After providing consent, they were instructed to read carefully through a summary of a criminal court case, and to imagine as if they were the judge overseeing the trial. After the case summary, the dependent measures were presented followed by several manipulation checks, validated inventories, and supplemental questions. Finally, participants provided demographic information including age, gender, political affiliation, and income.

## Results

### Hypothesis tests

#### (H1) Were prison sentence recommendations decreased when evidence for the defendant’s disorder was described as neurobiological as compared to psychological? (H2) Was this effect greater when the disorder was treatable?

Revised prison sentence recommendations were subjected to a two-way Analysis of Variance with two mental health status conditions (neurobiological, psychological) and two treatability status conditions (treatable, untreatable). There was a main effect of mental health status on prison sentencing, *F*(1, 219) = 13.07, *p* < .001, η_p_^2^ = .056, (Partial eta-squared effect sizes are interpreted using the following benchmark values, suggested by Richardson [35]: .0588 ≤ medium < .1379.) indicating, as predicted, that when neurobiological evidence was given as an explanation for the underlying disorder, participants recommended significantly shorter sentences (*M* = 0.95, *SE =* 0.14) than when psychological evidence was given (*M* = 1.65, *SE =* 0.14). The same pattern of results was found using the change in prison recommendation, *F*(1, 219) = 6.18, *p* = .014, η_p_^2^ = .027, as well as when subjected to a one-way ANOVA including the healthy condition, *F*(2, 327) = 33.64, *p* < .001, η^2^ = .171. Pairwise comparisons (Fisher’s LSD) showed that, as predicted, the recommended prison sentence was significantly shorter when the defendant had a disorder supported by neurobiological evidence (*M* = 0.95, *SE =* 0.13, *p* < .001) or psychological evidence (*M* = 1.65, *SE =* 0.13, *p* < .001), than when the defendant was healthy (*M* = 2.49, *SE =* 0.14). Similarly, the disorder supported by neurobiological evidence garnered significantly shorter prison sentences than the disorder supported by psychological evidence, *p* < .001. To assess whether the introduction of any health related information altered prison sentences, a two-tailed paired t-test was conducted within the healthy condition before and after disclosure that the defendant was, in fact, in good mental health. As anticipated, there was a null effect, *t*(106) = 1.46, *p* = .15, indicating that participants likely assumed that the defendant was of sound mental health, by default, before any evidence was presented.

As expected, there was a main effect of treatability status on prison sentencing, *F*(1, 219) = 7.18, *p* = .008, η_p_^2^ = .032, indicating that when participants were told the defendant’s disorder was treatable, they recommended significantly shorter prison sentences (*M* = 1.04, *SE =* 0.14) than when participants were told the disorder was untreatable (*M* = 1.56, *SE =* 0.14). However, there was no significant interaction between the mental health status presented and the treatability of the disorder, *F*(1, 219) < 0.001, *p* = .99, η_p_^2^ < .001. The same pattern of results was found using the change in prison recommendation, *F*(1, 219) = 13.95, *p* < .001, η_p_^2^ = .060 and *F*(1, 219) = 0.073, *p* = .79, η_p_^2^ < .001, respectively. As a whole, these results were consistent with H1 but not H2. See Table 1 for sentencing recommendations by condition.

**Table 1:** Revised prison sentence and involuntary hospitalization recommendations as a function of mental health status and treatability.

		Psychological		Neurobiological	
Treatability	Measure	*n*	*M (SE)*	*n*	*M (SE)*
**High**	Revised Prison Sentence (yrs.)	55	1.39 (.19)	57	0.69 (.19)
	Involuntary Hospitalization Term (yrs.)		1.27 (.17)		1.39 (.17)
**Low**	Revised Prison Sentence (yrs.)	54	1.91 (.20)	57	1.21 (.19)
	Involuntary Hospitalization Term (yrs.)		2.02 (.18)		2.59 (.17)

*Note*. *n = sample size*, *M =* mean, *SE =* standard error

#### (H3) Were involuntary hospitalization terms increased when evidence for the defendant’s disorder was described as neurobiological as compared to psychological? (H4) Was this effect greater when the disorder was untreatable?

Recommended involuntary hospitalization terms were subjected to a two-way Analysis of Variance with two mental health status conditions (neurobiological, psychological) and two treatability status conditions (treatable, untreatable). There was a main effect of mental health status on recommended involuntary hospitalization terms, *F*(1, 219) = 4.07, *p* = .045, η_p_^2^ = .018, indicating, as predicted, that when neurobiological evidence was given as an explanation for the defendant’s underlying disorder, participants recommended significantly longer recommended involuntary hospitalization terms (*M* = 1.99, *SE =* 0.12), than when psychological evidence was given (*M* = 1.65, *SE =* 0.12). Participants’ change in prison sentence and involuntary hospitalization terms were negatively correlated, *r*(223) = -.38, *p* < .001, suggesting a perceived trade off between these decision outcomes. The same pattern of results was found when recommended involuntary hospitalization terms were subjected to a one-way ANOVA, including the healthy condition, *F*(2, 327) = 42.99, *p* < .001, η^2^ = .208. Pairwise comparisons showed that, as predicted, the recommended involuntary hospitalization term was significantly longer when the defendant’s disorder was described neurobiologically (*M* = 1.99, *SE* = 0.12, *p* < .001) as well as when the evidence was described psychologically, (*M* = 1.64, *SE* = 0.12, *p* < .001), than when the defendant was healthy, (*M* = 0.46, *SE* = 0.12). Similarly, neurobiological evidence garnered significantly longer recommended involuntary hospitalization terms than psychological evidence, *p* = .040.

**Fig 1:**
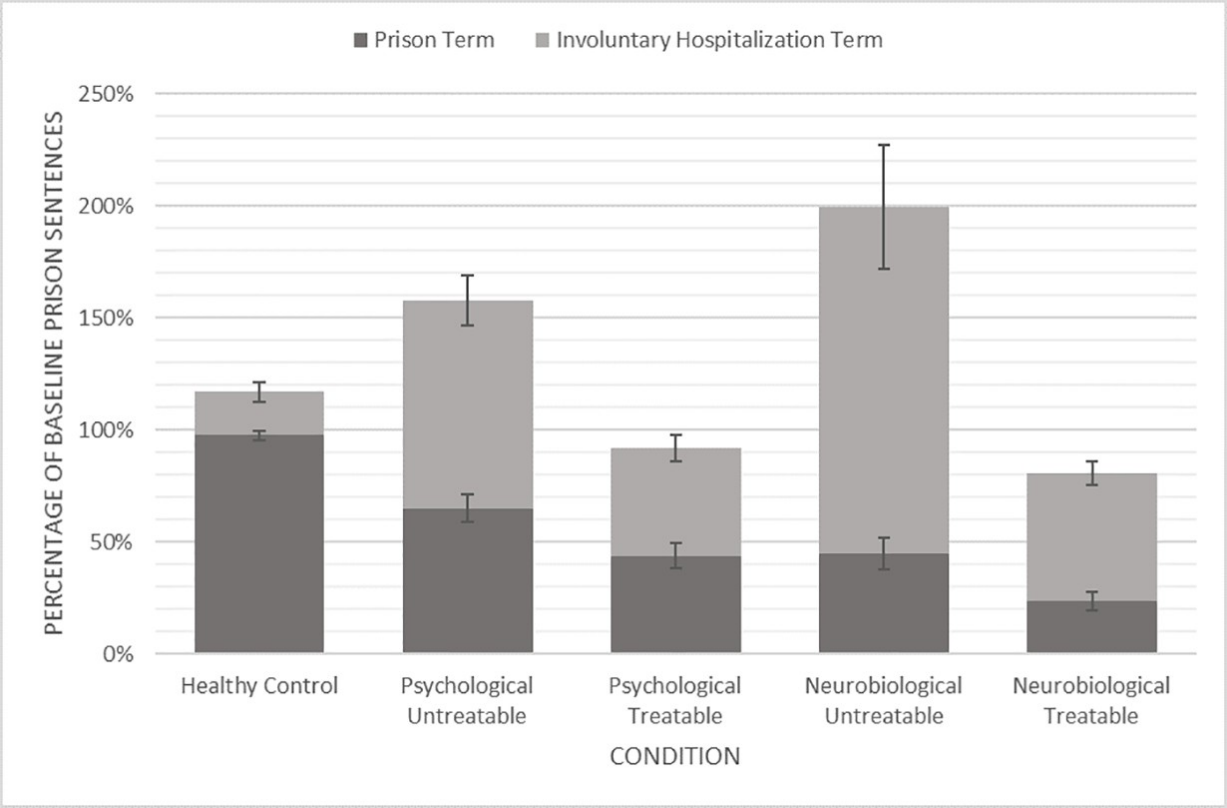
Punishment change score by condition. Bars denote the percentage change in time from individual baseline punishment rating across conditions, for their revised prison recommendation (dark grey) and recommendation for involuntary hospitalization (light grey). Statistically significant differences mirror the patterns described in H1-H4. Standard error bars shown.

As expected, there was also a main effect of treatability status on recommended involuntary hospitalization terms, *F*(1, 219) = 31.97, *p* < .001, η_p_^2^ = .127, indicating that when participants were told the defendant’s disorder was untreatable, they recommended significantly longer involuntary hospitalization terms (*M* = 2.31, *SE =* 0.12) than when participants were told the disorder was treatable (*M* = 1.33, *SE =* 0.12). However, there was no significant interaction between the mental health status presented and the treatability of the disorder, *F*(1, 219) = 1.69, *p* = .20, η_p_^2^ = .008. These results were consistent with H3 but not H4. See Fig 1 for punishment change scores by condition.

#### (H5a) Was the effect of mental health status on prison sentence recommendation best accounted for by reductions in deontological concerns rather than increases in consequentialist concerns?

We clustered the above items (Cronbach’s alpha > 0.70) into two categories in accordance with jurisprudence theories of punishment: deontological concerns (concerns about duty, such as the perceived obligation to punish offenders based on their moral blameworthiness) and consequentialist concerns (concerns about outcomes, such as the desire to punish to protect public safety). Items comprising the deontological factor were: the offender’s moral wrongness, moral responsibility, blameworthiness, desert of punishment, control of action, and free will (Cronbach’s α = .86). Items comprising the consequentialist factor were: the defendant’s dangerousness to society and likelihood of committing future crimes (α = .77). These clusters were confirmed in a two-factor solution identified by a principal components analysis of all items with varimax rotation, resulting in two independent factors (eigenvalues > 1) that matched our *a priori* grouping and explained 66.24% of the variance. We then used an ordinary least squares path analysis to examine whether these two types of concerns could account for, the observed effect of mental health status (neurobiological or psychological) on prison sentence term. The two composites were entered into a parallel regression model in order to compare their relative impact.

**Fig 2:**
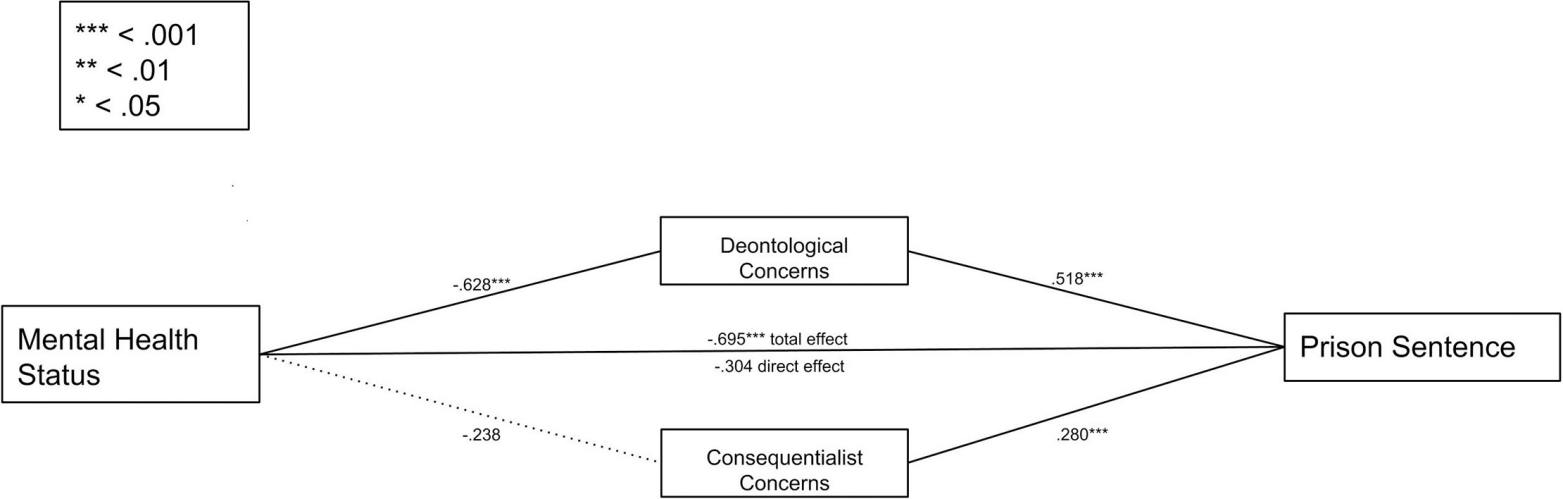
Regression coefficients for the relationship between mental health status and prison sentence as explained by deontological concerns and consequentialist concerns. Solid bold lines denote a significant relationship.

One third of the variance in recommended prison sentence length was explained by our parallel model (*R*^*2*^ = .33). The mitigating effect of neurobiological evidence was fully accounted for by deontological concerns (See Fig 2). As predicted, the neurobiological condition was a significant negative predictor of deontological concerns, *b* = -0.63, *SE* = 0.14, *p* < .001, and deontological concerns were a significant predictor of the prison sentence recommended to the defendant, *b* = 0.52, *SE* = 0.093, *p* < .001. A bootstrap confidence interval for the indirect effect of mental health status as explained by deontological concerns on prison sentence, *b* = -0.33, *SE* = 0.088, based on 5,000 samples, was entirely below zero (-0.52 to -0.17). The direct effect of mental health status on prison sentence was not significant, *b* = -0.30, *SE* = 0.17, *p* = .081. An identical pattern was observed for the effect of mental health status on the change in a participant’s prison sentence recommendation pre- and post-mental health status manipulation.

Consequentialist concerns also significantly predicted prison sentences, *b* = 0.28, *SE* = 0.071, *p* < .001, but mental health status did not predict consequentialist concerns, *b* = -0.24, *SE* = 0.18, *p* = .18. A bootstrap confidence interval for the indirect effect of mental health status as explained by consequentialist concerns on prison sentence, *b* = -0.067, *SE* = 0.053, included zero (-0.18 to 0.030), indicating that consequentialist concerns did not account for the effect of mental health status on prison sentence recommendation, consistent with our prediction. The observed regression model indicates that the mitigating effect of neurobiological evidence on prison sentence length can be explained by changes in deontological concerns—namely that the defendant was seen as less responsible for his criminal act.

**(H5b)** A test of the effect of deontological and consequentialist concerns on the relationship between mental health evidence and recommended involuntary hospitalization was not justified because no direct effect of evidence type on recommended involuntary hospitalization was observed.

#### EH1: Was the effect of mental health status on sentence length moderated by (a) counterfactual reasoning traits or (b) emotion regulation ability?

Change in prison sentence recommendation was subjected to a two-way Analysis of Variance with two mental health status conditions (neurobiological, psychological) and two counterfactual reasoning levels (low, high) as determined by a median split. A median split was performed to circumvent problems of multicollinearity observed using a more conventional, linear regression method. Contrary to expectation, there was no main effect of counterfactual reasoning level, *F*(1, 181) = 3.29, *p* = .071, η_p_^2^ = .018, and no interaction, *F*(1, 181) = 1.42, *p* = .23, η_p_^2^ = .008, indicating that the effect of mental health status on prison sentence length did not depend on a participants’ tendency to reason counterfactually.

Similarly, change in prison sentence recommendation was subjected to a two-way Analysis of Variance with two mental health status conditions (neurobiological, psychological) and two emotional regulation ability levels (low, high) as determined by a median split. Again, there was no main effect of emotional regulation level, *F*(1, 212) = 0.01, *p* = .93, η_p_^2^ < .001, and no interaction, *F*(1, 212) = 0.26, *p* = .61, η_p_^2^ = .001, indicating that the effect of mental health status on prison sentence length did not depend on a participants’ ability to regulate their emotions.

### Additional exploratory analyses

Several exploratory measures were examined to help contextualize and explain the results of our hypothesis tests.

#### Were deontological concerns reduced when the defendant’s disorder was described as neurobiological compared to psychological?

We theorized that any mitigating effect of neurobiological explanation on prison sentences would be driven primarily by deontological sentiments that the defendant should be held less morally responsible for the crime. If so, then participants presented with neurobiological evidence should likewise rate that defendant lower on measures of responsibility, blameworthiness, deservingness of punishment, free will, ability to stop himself from performing the crime, and higher on measures of trustworthiness. In this view, participants presented with neurobiological evidence should also perceive the crime as less characteristic of the defendant. All of these predictions were supported.

There was a main effect of mental health status on perceptions of the defendant’s moral responsibility for his crime, *F*(1, 219) = 16.13, *p* < .001, η_p_^2^ = .069, his blameworthiness, *F*(1, 219) = 15.09, *p* < .001, η_p_^2^ = .064, his deservingness of punishment, *F*(1, 219) = 12.26, *p* = .001, η_p_^2^ = .053, free will, *F*(1, 219) = 21.89, *p* < .001, η_p_^2^ = .091, ability to stop himself from committing the crime, *F*(1, 219) = 10.58, *p* = .001, η_p_^2^ = .046, his trustworthiness, *F*(1, 219) = 7.82, *p* = .006, η_p_^2^ = .034, and the perception that the defendant’s action was an expression of his essential character (i.e., his “deep self”), *F*(1, 219) = 20.91, *p* < .001, η_p_^2^ = .087. Those in the neurobiological condition saw the defendant as less morally responsible (see Table 2 for exact group values), less blameworthy, less deserving of punishment, having less free will, less able to stop himself from committing the crime, more trustworthy, and perceived the crime as less characteristic of the defendant than those in the psychological condition.

**Table 2:** Deontological and consequentialist concerns as a function of mental health status and treatability status.

	Psychological	Neurobiological	Psychological	Neurobiological
Treatability	*M (SE)*	*M (SE)*	*M (SE)*	*M (SE)*
	Moral Responsibility		Action an Expression of Defendant's Character	
**High**	6.22 (0.18)	5.53 (0.18)	4.96 (0.21)	3.97 (0.20)
**Low**	6.28 (0.18)	5.54 (0.18)	5.02 (0.21)	4.16 (0.20)
	Blameworthiness		Danger to Society	
**High**	5.98 (0.18)	5.23 (0.17)	5.09 (0.17)	4.33 (0.17)
**Low**	6.06 (0.18)	5.44 (0.17)	6.13 (0.17)	6.21 (0.17)
	Deserving of Punishment		Likelihood of Reoffense	
**High**	6.02 (0.18)	5.16 (0.17)	3.93 (0.17)	3.39 (0.17)
**Low**	6.00 (0.18)	5.63 (0.17)	4.98 (0.18)	5.23 (0.17)
	Free Will		Perceived Efficacy of Treatment	
**High**	5.38 (0.21)	4.18 (0.21)	3.22 (0.14)	3.79 (0.13)
**Low**	5.28 (0.21)	4.52 (0.21)	1.72 (0.14)	1.46 (0.13)
	Ability to Stop Himself		Perceived Impact of Evidence	
**High**	4.82 (0.21)	3.97 (0.21)	2.38 (0.14)	1.97 (0.14)
**Low**	4.69 (0.22)	4.16 (0.21)	3.11 (0.14)	2.53 (0.14)
	Trustworthiness		Importance of Exam Results	
**High**	2.29 (0.17)	3.18 (0.16)	3.64 (0.16)	4.32 (0.16)
**Low**	2.09 (0.17)	2.12 (0.16)	3.17 (0.17)	4.00 (0.16)

*Note*. *M =* mean, *SE =* standard error

In contrast, there was no main effect of treatability status on any of these measures except trustworthiness, *F*(1, 219) = 14.63, *p* < .001, η_p_^2^ = .063, such that when the defendant’s disorder was treatable he was perceived as more trustworthy than when the disorder was untreatable. Similarly, there was no interaction effect between mental health status and treatability on any measure besides trustworthiness, *F*(1, 219) = 6.82, *p* = .010, η_p_^2^ = .030, such that the positive effect of treatability on trustworthiness was larger in the neurobiological condition, *p* < .001, η_p_^2^ = .063. As might be expected, this effect did not extend to the condition in which the disorder was described as untreatable, *p* = .90, η_p_^2^ < .001.

#### Were consequentialist concerns increased when the defendant’s disorder was described as neurobiological compared to psychological?

We theorized that any aggravating effect of the neurobiological explanation on involuntary hospitalization would be motivated primarily by concerns of the defendant’s future danger to society. If so, then participants presented with neurobiological evidence should characterize that defendant as more dangerous. However, this prediction was not supported. Instead, a main effect of mental health status on the defendant’s dangerousness, *F*(1, 219) = 4.05, *p* = .045, η_p_^2^ = .018, indicated that participants presented with neurobiological evidence found the defendant *less* dangerous than those presented with psychological evidence. This effect was further supported by an interaction, *F*(1, 219) = 6.22, *p* = .013, η_p_^2^ = .028, in which the mitigating effect of treatability, *F*(1, 219) = 75.23, *p* < .001, η_p_^2^ = .256, on perceived dangerousness was larger when the evidence was described as neurobiological compared to psychological, *p* = .002, η_p_^2^ = .045. Pairwise differences were not found between neurobiological and psychological evidence in the untreatable condition, *p* = .74, η_p_^2^ = .001. One interpretation of these counter-intuitive results is that participants perceived involuntary hospitalization not as a way to incapacitate morally culpable people that pose a danger to society but as a way to provide medical attention to those most in need of it, including, potentially, those who pose a danger to *themselves*—a distinction that our dangerousness measure may not have captured.

#### Was the treatment for the defendant’s condition perceived as more efficacious when that condition was described as neurobiological versus psychological?

If the aggravating effect of neurobiological explanation on recommended involuntary hospitalization term was not due to perceptions of increased dangerousness, perhaps it could be due to a perception that neurobiological disorders are more treatable than psychological disorders, at least in inpatient contexts. If so, then people should rate the treatment of the neurobiological disorder as more efficacious. We observed partial support for this hypothesis. There was a significant interaction between mental health status and treatability, *F*(1, 219) = 9.64, *p* = .002, η_p_^2^ = .042, such that participants presented with neurobiological evidence of a treatable disorder expressed stronger belief in the efficacy of the treatment than those presented with psychological evidence of a treatable disorder, *p* = .003, η_p_^2^ = .040. As might be expected, this effect did not extend to the condition in which the disorder was described as untreatable, *p* = .17, η_p_^2^ = .009. Moreover, there was no main effect of mental health status on the efficacy of the defendant’s treatment, *F*(1, 219) = 1.28, *p* = .26, η_p_^2^ = .006.

#### Were neurobiological descriptions of the defendant’s disorder seen as more important than psychological descriptions?

Next we examined whether participants expressed explicit attitudes consistent with the mitigating effect of neurobiological evidence on punishment. If so, this would support the interpretation that participants were consciously aware of the reasons driving their decision. To address this question, participants indicated the extent to which they thought the evidence of the defendant’s condition “decreases, increases, or has no effect on” their initial punishment. They were also asked how important the exam results were to their punishment decision.

As expected, participants given neurobiological evidence reported the evidence as more mitigating and more important than those given psychological evidence, *F*(1, 219) = 12.42, *p* = .001, η_p_^2^ = .054; *F*(1, 219) = 21.78, *p* < .001, η_p_^2^ = .090. Similarly, participants told that the disorder was treatable reported the evidence as more mitigating and important than those told the disorder was untreatable, *F*(1, 219) = 20.62, *p* < .001, η_p_^2^ = .086; *F*(1, 219) = 5.87, *p* = .016, η_p_^2^ = .026. There were no significant interactions, *F*(1, 219) = 0.35, *p* = .56, η_p_^2^ = .002; *F*(1, 219) = 0.23, *p* = .64, η_p_^2^ = .001.

## Discussion

The purpose of this project was to investigate the effect of brain-based evidence of an impulse control disorder on lay sentencing judgments. We observed three key findings: (1) Both brain evidence and psychological evidence had mitigating effects on prison sentences, but the mitigating effect of brain evidence was stronger. (2) Yet that same brain evidence evoked relative increases in involuntary hospitalization terms. (3) The variation in sentencing judgments was best explained by deontological considerations pertaining to moral culpability.

These findings suggest that lay people assign more importance to mental health evidence whose causes are described in neurobiological terms than in psychological terms. As predicted, this evidence seems to both favor or disfavor the defendant depending on the decision type: Although evidence of a neurobiological cause of a disorder may mitigate prison punishment, the same evidence can place the defendant at an increased risk of involuntary hospitalization. Though the effect sizes of our primary hypotheses were not large, they are still potentially relevant to the law, where punishment practices and policies can have far-reaching consequences for society when deployed over large temporal and geographic scales.

One plausible explanation for this effect is that neurobiological evidence primes fact-finders to preferentially attend to the distinctly physical causes of behavior, and this feeds their intuitions that the behavior is outside the defendant's control. Perceptions of reduced control may, in turn, reduce attributions of responsibility while potentially increasing the belief that the defendant requires medical intervention (see [26]).

The mitigation effect is consistent with the operation of a deontological motive for punishment, namely that punishment should be proportionate to the offender’s moral culpability. The reason for the aggravating effect of neurobiological evidence on recommended involuntary hospitalization term is less clear. “Double edge” theories would explain this increase using consequentialist reasons such as a desire to protect society from danger or a desire to provide treatment to those who would most benefit from it, but the neurobiologically disordered defendant was rated as no more dangerous or treatable than the other disordered defendant. This leaves open the question of exactly why people assigned more hospitalization time to the neurobiologically disordered defendant. We speculate that the answer hinges on how people interpret the specific purpose of involuntary hospitalization. Perhaps, for instance, people considered involuntary hospitalization more justified for long-term disease management, even if their treatment prospects are low. Similarly, people in this condition might have felt a greater *obligation* to provide care, regardless of treatment prospects. Our manipulation checks did not make such fine distinctions. If these interpretations are confirmed in future research, they would be compatible with “moral education theory,” the idea that punishments may be justified, or perhaps even obligatory, to the extent that they benefit to the person being punished [36]. Alternatively, involuntary hospitalization could be used to quarantine people perceived to be ill-fit for society. This interpretation is consistent with previous research suggesting that people feel the need to socially distance themselves from individuals with biologically described mental disorders [37–40]. In such cases, increased hospitalization time for defendants in the neurobiological condition should be understood as “aggravating” only in the narrow sense that it was defined as involuntary, but not in a classically retributive sense.

This study is the first to quantitatively dissociate the divergent effects of neurobiological evidence on sentencing decisions (i.e., the “double-edged sword”). In a similar vein, research by Aspinwall et al. [7] and Fuss et al. [18], found that while biological explanations for a crime mitigated punishment recommendations or estimations of legal responsibility, some increased consideration of future dangerousness and support for involuntary commitment was found. However, in those studies, this support was observed by qualitative measures only. The present study validated this effect using quantitative measures. Further, the dependent measures in our study allowed participants to award prison time and involuntary hospitalization time separately. This approach was employed to distill the moralistic punitive motives from incapacitative and treatment-based motives, and could explain why our study uniquely observed the predicted double-edge effect.

### Limitations and future directions

As with all studies, our findings are necessarily limited by our procedural choices. We included an alternative measure to prison punishment (involuntary hospitalization) to distill the nature of participants punitive motives. Even so, involuntary hospitalization itself can be used for a variety of purposes that we could not disentangle, such as treatment, incapacitation, or possibly even punishment. Efforts to understand participants motivations for such decisions should consider a wider array of punishment measures designed to fulfill distinct aims or should devise additional manipulations that achieve this effect.

It is also unclear why participant’s individual differences (i.e., the ability to reason counterfactually and the ability to regulate one’s own emotions) did not explain individual susceptibility to change in prison sentence. It is possible that this was a consequence of weak construct validity. However, these results are consistent with previous literature showing that those high in cognitive ability are no less susceptible to cognitive biases (and in some cases, more susceptible) than those low in cognitive ability [28]. Future studies should address these possibilities using other theoretically motivated individual difference measures.

This study investigated punishment judgments in an Internet-based lay sample and do not necessarily generalize to legal samples such as judges and jurors or to the broader U.S. population. Future research on lay samples should aspire to full randomization across key dimensions including geography and political party affiliation. Likewise, this research should be extended to legal samples in attempt to replicate these effects among groups whose judgments are directly consequential for criminal defendants, such as trial court judges. Lastly, it would be helpful to move beyond experimental survey methods and into more realistic presentation modalities, such as mock trials, to establish greater ecological validity.

Our study design did not permit investigation of potential interactive relationships between psychological and neurobiological evidence. In real criminal trials, both types of evidence might be presented together. Inclusion of a combined condition would address whether their joint presentation might have multiplicative, or perhaps antagonistic, effects on attributions of responsibility and punishment.

Finally, interpretation of evidence likely depends on the type of crime and mental health condition portrayed. Our vignettes described a sexual assault in order to increase the plausibility of the use of involuntary hospitalization, but this choice departs from other studies in this body of literature. Likewise, the defendant’s mental condition was defined as an “impulse control disorder.” This decision was made to minimize unknown preconceptions about culturally loaded labels such as psychopathy, schizophrenia, and psychosis, thus differentiating this study from others of its kind [7, 8, 21]. Future studies should consider controlling these cross-study differences or consider other theoretically-motivated causes of behavior including prototypically physical disorders (e.g., bipolar disorder) as well as prototypically psychological disorders (e.g., adjustment disorder).

Limitations notwithstanding, these findings are important for criminal law procedure, and particularly for policy makers, because they highlight a potential contextual effect that has not been examined in previous research. Specifically, policy makers must confront the question of how to manage the effects that we observed. For example, when neuroscientific evidence is introduced to support mental illness arguments, should it be accompanied *pro forma* by information about its potentially biasing effects? Should it be accompanied by information about the defendant’s amenability to treatment? When may neuroscience evidence stand alone, and when must it be accompanied by corresponding behavioral evidence? Should judges be required to receive legal education on neuroscience evidence? Should jurors be entitled (or required?) to review the treatment options or mandates that would apply if the defendant is excused on grounds of mental illness? Additional scholarship is needed to examine these and other practical applications of this research.

## Supporting information

S1 Appendix Experimental stimuli. (DOCX)

S1 Dataset Data underlying findings. (CSV)
