# Supplementary material for: Reconciling the opposing effects of neurobiological evidence on criminal sentencing judgments
Source: PLoS One. 2019 Jan 18;14(1):e0210584. doi: 10.1371/journal.pone.0210584 (PMC6338372; doi:10.1371/journal.pone.0210584)
Supplement: S1 Appendix — (DOCX) [file pone.0210584.s001.docx]

## **Appendix**

Experimental Stimuli

*NEURO / PSYCHOLOGICAL: The [neurologists / psychologists] examined the defendant by conducting [magnetic resonance imaging scans of the defendant’s brain / a series of clinical interviews with the defendant] to look for any evidence of a [neurological / mental] disorder. Each [neurologist / psychologist] independently determined that the defendant has [a large tumor in a part of the brain involved in impulse control / an impulse control disorder]. The [neurologists / psychologists] agreed that this [tumor / disorder] could explain his impulsive criminal behavior.*

*NEURO TREATABLE / UNTREATABLE: During this time, Mr. Edwards underwent neurosurgery in an attempt to remove his brain tumor. [The surgeon was able to fully remove it without damaging / However, because of the nature of the tumor, the surgeon was unable to fully remove it because doing so would cause severe damage to the] healthy brain tissue. Upon completion of the three-month discovery period, Mr. Edwards’ neurologists testified that he [is showing real improvement. He no longer acts impulsively./ has not shown any improvement. He continues to act impulsively.] His neurologists maintain that he [should be classified as LOW RISK for future impulsive criminal behavior on the condition that he continues to attend his weekly doctor visits to monitor for tumor re-growth / is not expected to recover and so should be classified as HIGH RISK for future impulsive criminal behavior].*

*PSYCHOLOGICAL TREATABLE / UNTREATABLE: During this time, Mr. Edwards was enrolled in an intensive cognitive-behavioral therapy program for individuals with impulse control problems. [However] Upon completion of the three-month discovery period, Mr. Edwards’ psychologists testified that he [is showing real improvement. He no longer acts impulsively / has not shown any improvement. He continues to act impulsively.] His psychologists maintain that he [should be classified as LOW RISK for future impulsive criminal behavior on the condition that he continues to attend his weekly therapy sessions to monitor for behavior changes / is not expected to recover and so should be classified as HIGH RISK for future impulsive criminal behavior].*

*HEALTHY: The experts examined the defendant. Each expert independently determined that the defendant did not have any mental health issues and concluded that his mental health status was not a relevant factor in his crime.*

*Given this new information, Mr. Edwards is eligible to be considered for a sentencing review.*

*You may now choose among either or both of two options:*

*(1) You may change the length of Mr. Edwards’ prison sentence or keep it the same.*

*(2) You may also sentence Mr. Edwards to any amount of time in an Inpatient Hospital.*

*Inpatient Hospitals are secured facilities for dangerous offenders who have serious mental or*

*behavioral conditions. The purpose of Inpatient Hospitals is to treat and rehabilitate offenders*

*rather than punish them.*

Revised Prison Recommendation: *Given his sexual assault conviction, how many years should Mr. Edwards serve in prison?* (0-4 yrs.)

Involuntary Hospitalization Recommendation: *How many years should Mr. Edwards serve in an inpatient hospital?* (0-4 yrs.)

EXPLORATORY MEASURES. (All scales labels were *Strongly Disagree* (1) to *Strongly Agree* (7) unless otherwise noted.)

Moral Responsibility: *Mr. Edwards should be held morally responsible for his crime.*

Blameworthiness: *Mr. Edwards is blameworthy for the crime that he committed.*

Desert of Punishment: *Mr. Edwards deserves* *to be punished for his crime.*

Free Will: *When Mr. Edwards assaulted the neighbor, he was acting of his own free will.*

Ability to Stop Himself: *Mr. Edwards could have stopped himself from committing the crime.*

Trustworthiness: *Mr. Edwards is a trustworthy person.*

The Crime was an Expression of His Character: *Mr. Edwards’ criminal act was an expression of his essential character.*

Danger to Society: *Mr. Edwards is a danger to society.*

Likelihood of Reoffense: *Mr. Edwards will commit another crime within 3 years of his release.*

Perceived Efficacy of Treatment: *How effective do you think the treatment was for Mr. Edwards?*

Perceived Impact of Evidence: *To what extent do you think the evidence of Mr. Edwards’ condition decreases, increases, or has no effect on your initial punishment? (Greatly Decreases (1)* to *Greatly Increases (5))*

Importance of Exam Results: *How important were the experts’ exam results in your punishment decisions? (Not Important (1)* to *Very Important (5))*
